# Clinical value of thyroid related hormones combined with neutrophil to lymphocyte ratio in patients with nonalcoholic fatty liver disease

**DOI:** 10.1097/MD.0000000000031978

**Published:** 2022-12-23

**Authors:** Ping Li, Liang Liu, Bin Niu, YuQiang Mi, YongGang Liu, Jing Feng, Peng Zhang, Xue Wu, WeiKe Chu

**Affiliations:** a Clinical School of the Second People’s Hospital, Tianjin Medical University, Tianjin, China; b Department of Hepatology, Tianjin Second People’s Hospital, Tianjin, China; c First Hospital Affiliated to Nanchang University, Nanchang, China; d School of Graduates, Tianjin Medical University, Tianjin, China.

**Keywords:** fibrosis, liver biopsy, neutrophil to lymphocyte ratio, nonalcoholic fatty liver disease, noninvasive evaluation, thyroid related hormones

## Abstract

In sufferers with nonalcoholic fatty liver disease (NAFLD), the differences of thyroid associated hormones and neutrophil to lymphocyte ratio (NLR) in different liver pathological groups have been compared.

**Methods:**

Patients with NAFLD diagnosed by liver biopsy in our hospital from July 2012 to February 2019 were selected. All subjects were divided into nonalcoholic steatohepatitis (NASH) team and non-NASH group, no/mild fibrosis group (F0-1) and significant fibrosis group (F2-4). The differences of thyroid related hormones and NLR in these groups were in contrast, respectively. For the TSH, we conducted further evaluation based on gender.

**Results:**

The TSH and NLR in NASH patients were significantly higher than non-NASH patients, but there was no considerable difference in free triiodothyronine (FT3) and free thyroxine (FT4) between the 2 groups. In the gender-based subgroup analysis, the variations of TSH between the 2 groups were nonetheless statistically significant (*P* < .05). The TSH and NLR in the significant fibrosis group were higher than these in the non/mild liver fibrosis group, and the differences were statistically significant (*P* < .05), but there was no large difference in FT3 and FT4 between the 2 groups (*P* > .05). In addition, in the gender-based subgroup analysis and further multivariable analysis, the variations of TSH between the 2 groups were still statistically significant (*P* < .05).

**Conclusions:**

In this study, we found that serum thyroid stimulating hormone (TSH) and neutrophil to lymphocyte ratio (NLR) were closely associated to the severity of NAFLD, suggesting that this simple available laboratory index may additionally be incorporated into the future noninvasive diagnostic scoring model to predict the incidence of NASH and the degree of fibrosis.

## 1. Introduction

Nonalcoholic fatty liver disease (NAFLD) is the most prevalent liver disease in the world, accounting for about 25% of the whole population.^[1]^ With the enhancement of living standards, the prevalence of NAFLD is additionally increasing year by year.^[2]^ Studies have shown that the prevalence of NAFLD in obese people is about 75% to 100%.^[3]^ The rising incidence of diabetes and obesity will lead to an increase in the incidence of issues of nonalcoholic steatohepatitis (NASH), liver cirrhosis and end-stage liver disease.^[4]^ At the same time, due to the lack of effective treatment, NAFLD has become the most common chronic liver disease in China. NAFLD is a clinicopathological syndrome whose disease spectrum ranges from hepatic steatosis and NASH to liver cirrhosis and hepatocellular carcinoma.^[5]^ Compared with hepatocyte steatosis, NASH is more likely to develop into liver cirrhosis and liver cancer.^[6]^

Thyroid stimulating hormone (TSH) is a hormone secreted by the pituitary gland, which causes the secretion of thyroid hormone (TH) by performing on the thyroid gland. Previous studies have shown that in addition to organic effects by regulating the stage of TH, serum TSH also has a direct regulatory effect on liver lipid metabolism.^[7]^TH may also be a risk factor for the pathophysiology of NAFLD and its progression to liver fibrosis. The level of TH is also concerned in blood glucose and lipid metabolism, as well as insulin resistance.^[8]^

Recent studies have shown that hypothyroidism is closely associated with the severity of chronic liver disease. In patients with NASH, the development of fibrosis was negatively correlated with serum thyroid hormone levels and the expression of thyroid regulatory genes such as thyroid hormone responsive protein (*THRSP*) in the liver..^[9]^ In addition, the degrees of free triiodothyronine (FT3) and free thyroxine (FT4) were significantly decreased in patients with liver cirrhosis, and the level of FT3 was once negatively correlated with Child-Pugh score, that is, the level of FT3 was decrease in progressive diseases.^[10]^ A recent large population-based prospective cohort consisting of 9419 participants confirmed these findings and similarly found that those with hyperthyroidism can prevent the progression of NAFLD.^[11]^ In addition, another cross-sectional study confirmed that elevated serum TSH levels were independent predictors of NASH and fibrosis in NAFLD patients with normal thyroid function. The study involved 425 NAFLD subjects from South Korea who underwent biopsies, and the study further suggested a relationship between elevated serum TSH levels and NASH and its fibrosis within the normal range.^[12]^ More importantly, these findings have been further confirmed in a multi-ethnic study, in which the researchers surveyed 7259 American adults who had available thyroid function laboratory data and relevant data used to calculate NAFLD fibrosis scores.^[13]^

However, the research on the relationship between thyroid related hormones and NASH is still controversial.^[9,14,15]^ In addition, the outcomes of the recent meta-analysis on the association between thyroid-related hormones and NAFLD are now not consistent.^[16,17]^ At the same time, there are few studies to explore the relationship between thyroid-related hormones and the severity of NAFLD disease confirmed by biopsy.

In addition, oxidative stress and chronic inflammation play an important role in the development of NAFLD disease into NASH and liver fibrosis.^[18–20]^ Some studies have found that patients with NASH have higher levels of oxidized fatty acids and inflammatory cytokines than patients with easy hepatic steatosis.^[21,22]^ The ratio of neutrophils to lymphocytes (NLR) is obtained by simple calculation of neutrophil count (NC) and lymphocyte count (LC) in blood routine tests. It is an effortlessly available and inexpensive subclinical marker of inflammation. NLR integrates information about 2 different immune pathways: lymphocytes, which represent regulatory pathways, and neutrophils, which are responsible for persistent inflammation^[23,24]^ Therefore, NLR is an indicator of the overall inflammatory state of the body, and a higher ratio may also be found in NAFLD patients with more severe disease. Studies have shown that NLR has been used as a prognostic factor for infectious, inflammatory and malignant diseases.^[25]^ However, the conclusion of NLR in predicting liver injury in NASH and other chronic liver diseases is still controversial.

## 2. Materials and methods

### 2.1. Participants and study design

The study subjects selected 127 NAFLD patients diagnosed by liver pathological biopsy in our hospital from July 2012 to February 2019. Inclusion criteria were as follows: Age 18 years and above; The diagnosis of NAFLD complies with the “Guidelines for the Diagnosis and Treatment of nonalcoholic Fatty Liver Disease” (updated 2010 version and 2018 version),^[26,27]^ and all patients in the group underwent liver histopathological examination. Main exclusion criteria included: Excessive drinking history: male more than 30 g/d, female more than 20 g/d; Presence of liver disease caused by other etiologies (e.g., alcoholic liver disease, autoimmune hepatitis, drug-induced liver disease, Wilson disease, hemochromatosis, or a primary related diseases such as biliary cholangitis or primary sclerosing cholangitis); Recent usage of immunosuppressants, steroid hormones, chemotherapy drugs, thyroid hormones or antithyroid drugs that affect thyroid function, inflammation and immune cells; Endocrine system diseases affecting thyroid function (e.g., pituitary disease, thyroid diseases); Severe underlying diseases; During pregnancy and lactation.

All experiments involving liver tissues in this study were conducted in accordance with the Declaration of Helsinki and the relevant requirements of the hospital ethics committee, and informed consent was given from each subject.

### 2.2. Assessments and laboratory testing

General data of the included study subjects was collected, such as age, gender, body mass index (BMI), history of hypertension, and history of diabetes. After admission, the patient’s fasting 12-hour venous blood was taken in the morning to test its laboratory indicators: Japanese HITACH1 automatic biochemical analyzer-7180 (reagent from BECKMAN) was used to detect alanine aminotransferase (ALT) and aspartate aminotransferase (AST), γ-glutamyltranspeptidase (γ-GT), total cholesterol (TC), triglyceride (TG), high-density lipoprotein (HDL), low-density lipoprotein (LDL), fasting plasma glucose (FPG); use Japanese automatic blood cell analyzer SysmexXN-2000 (reagents purchased from Roche, Germany) for detection Platelet (PLT), NC, LC, and calculate the NLR value of each patient. The normal reference range for NC is (1.8–6.3) × 10^9^/L, and the normal reference range for LC is (1.1–3.2) × 10^9^/L; FT3, FT4, and TSH are detected by an automatic electro-chemiluminescence analyzer (reagents purchased from Roche, Switzerland). The normal reference range of FT3 is 3.10 to 6.80 pmol/L, and the normal reference range of FT4 is 12.0 to 22.0 pmol/L. The normal reference range of TSH is 0.27 to 4.20 mIU/L. The clinical inspection operations are carried out by professional and technical personnel in accordance with the operating instructions.

### 2.3. Liver histology

After informed notification before the operation, the patient was instructed to take the supine position, and after positioning for ultrasound, the liver pathological tissue specimen was obtained by percutaneous liver biopsy with a 16G puncture needle and fixed with 10% formalin. The length of each liver pathological tissue specimen was not less than 1.5 cm, and it contained no less than 6 portal areas under the microscope. After conventional dehydration and paraffin embedding, 4 μm serial sections were adhered to the glass slide for HE, reticular fiber and Masson staining. Two pathologists independently observed the image reading work. If the diagnosis was inconsistent, they read the image again and discussed it. The NAFLD activity score (NAS) system was used to assess the degree of hepatocyte steatosis (0 points: <5%; 1 point: 5%–33%; 2 points: 34%–66%; 3 points: >66%), lobular inflammation (0 points: no necrotic foci; 1 point: <2 necrotic foci; 2 points: 2–4 necrotic foci; 3 points: >4 necrotic foci), hepatocyte ballooning (0 points: No ballooning; 1 point: ballooning is rare; 2 points: ballooning is more common) The pathological scores were performed separately. The NAS score was the cumulative sum of the scores of hepatocyte steatosis, lobular inflammation, and hepatocyte ballooning. If it was greater than 4 points, it was diagnosed as NASH.^[28]^ In addition, liver fibrosis staging included F0: no fibrosis; F1: perisinus or periportal fibrosis; F2: perisinus and periportal fibrosis; F3: bridging fibrosis; F4: cirrhosis.^[28]^ At the same time, we defined fibrosis F2 to F4 confirmed by liver biopsy as significant fibrosis (moderate to severe fibrosis).

### 2.4. Statistical analysis

Counting data was expressed by frequency and composition ratio. When the measurement data conformed to the normal distribution, it was expressed by ‾X ± S, and when it does not conform to the normal distribution, it was expressed by M (P25, P75). The corresponding test method was chosen according to the type of data. The measurement data tested for normality first. The independent sample *t* test was used for the comparison of the mean difference between each 2 groups of normal distribution data, and the nonparametric test was used when it did not conform to the normal distribution. The Mann-Whitney U or Kruskal-Wallis H test; the comparison between the enumeration data groups used the ‾X2 test. Factors associated with changes in fibrosis were accessed using univariate and logistic regression analysis. Receiver operating characteristic (ROC) curve and calculating the area under the curve were used to evaluate the predictive value of TSH and TSH on pathological inflammation grading and fibrosis staging of liver. Statistical analysis was performed using SPSS 22, MedCalc 15.8 and GraphPad Prism 6 statistical software, and *P* < .05 was considered statistically significant.

## 3. Results

### 3.1. Characteristics of the included patients

A total of 127 NAFLD patients diagnosed by liver biopsy were enrolled in this study, with an average age of 43.37 ± 14.07 years old. Among them, 59 (46.5%) were males, with an average BMI of 26.91 ± 3.58 kg/m^2^. 31.5% and 19.7% of the patients were combined with hypertension and diabetes, respectively. The average of NC is 3.51 ± 1.29 × 109/L, the average of LC is 1.88 ± 0.65 × 109/L, the average of NLR is 2.14 ± 1.30; the average of FT3 is 4.95 ± 0.69 pmol/L, the average of FT4 level is 16.00 ± 2.05 pmol/L, and the average level of TSH is 2.48 ± 1.88 mIU/L. The results are shown in Table 1.

**Table 1:** The clinical characteristics of included patients.

Variables	Total patients (N = 127)
Age (yr)	43.37 ± 14.07
Gender (M/F)	59/68
Hypertension (No/Yes)	87/40
Diabetes (No/Yes)	102/25
BMI (kg/m^2^)	26.91 ± 3.58
ALT (U/L)	74 (32,139)
AST (U/L)	41 (25,73.13)
γ-GT (U/L)	72 (41,135)
TC (mmol/L)	4.97 ± 1.04
TG (mmol/L)	1.76 ± 1.26
HDL (mm/L)	1.11 ± 0.29
LDL (mmol/L)	2.85 ± 0.80
FPG (mmol/L)	6.01 ± 1.06
FT3 (pmol/L)	4.95 ± 0.69
FT4 (pmol/L)	16.00 ± 2.05
TSH (mIU/L)	2.48 ± 1.88
PLT (10^^9^/L)	229 (184,273)
NC (10^^9^/L)	3.51 ± 1.29
LC (10^^9^/L)	1.88 ± 0.65
NLR	2.14 ± 1.30

ALT = alanine aminotransferase, AST = aspartate aminotransferase, FPG = fasting plasma glucose, FT3 = free triiodothyronine, FT4 = free thyroxine, HDL = high-density lipoprotein, LC = lymphocyte count, LDL = low-density lipoprotein, NC = neutrophil count, NLR = neutrophil to lymphocyte ratio, TC = total cholesterol, TG = triglyceride, TSH = PLTplatelet, γ-GT = γ-glutamyltranspeptidase.

Table 2 describes the histopathological characteristics of all the study subjects, including liver cell steatosis, lobular inflammation, ballooning and liver fibrosis staging. The results showed that 67 patients (52.76%) had moderate to severe liver cell steatosis, 69 cases (54.33%) patients had moderate to severe lobular inflammation, 114 (89.76%) patients had hepatocellular balloon degeneration, 116 (91.34%) patients had some degree of fibrosis confirmed by liver biopsy, 22 cases (17.32%) Patients with advanced liver fibrosis (F3-4), 10 patients (7.87%) were diagnosed with liver cirrhosis (F4) by liver biopsy, histopathological features suggested signs of autoimmune hepatitis such as interface hepatitis, plasma Patients with cell infiltration and rosettes were excluded.

**Table 2:** Histopathological characteristics of the included subjects.

Variables	Total patients (N = 127)
Steatosis	
1	60 (47.2%)
2	44 (34.6%)
3	23 (18.1%)
Inflammation	
0	13 (10.2%)
1	45 (35.4%)
2	47 (37%)
3	22 (17.3%)
Ballooning	
0	13 (10.2%)
1	89 (70.1%)
2	25 (19.7%)
Fibrosis	
0	11 (8.7%)
1	40 (31.5%)
2	54 (42.5%)
3	12 (9.4%)
4	10 (7.9%)

### 3.2. Comparison of clinical and pathological features based on NASH and fibrosis groups

Among the study population included in this study, according to Brunt’s diagnostic criteria, there were 72 cases (56.69%) in the NASH group and 55 cases (43.31%) in the non-NASH group. Table 3 describes the general clinical characteristics of the 2 groups. The levels of BMI, ALT, AST, FPG, NC, NLR, and TSH in NASH patients were significantly higher than those of the non-NASH group. At the same time, there were significant differences in the distribution of gender, hypertension and diabetes between the NASH group and the non-NASH group. Among them, the NASH group There were 40 males (55.6%), 23 patients (31.9%) with hypertension, and 19 patients (26.4%) with diabetes, both of which were higher than those of the non-NASH group, with significant differences (all *P* < .05); There were no significant differences in age, γ-GT, TC, TG, HDL, LDL, PLT, LC, FT3, FT4 levels between the 2 groups (all *P* > .05).

**Table 3:** Comparison of general clinical data between NASH and non-NASH groups.

Variables	NASH (N = 72)	Non-NASH (N = 55)	Statistics	*P* value
Age (yr)	42.46 ± 14.87	44.56 ± 12.98	0.835	.405
Gender (M/ F)	40/32	19/36	5.533	.019
Hypertension (No/Yes)	49/23	38/17	7.248	.027
Diabetes (No/Yes)	53/19	49/6	13.18	.001
BMI (kg/m^2^)	27.73 ± 3.48	25.85 ± 3.45	−3.02	.003
ALT (U/L)	90(50.43,177.75)	45(21,104)	−3.798	<.001
AST (U/L)	57.25(33,89)	27(19,47)	−4.411	<.001
γ-GT (U/L)	73.25(46.25,140.75)	63(32,135)	−0.735	.462
TC (mmol/L)	4.97 ± 1.12	4.96 ± 0.93	−0.02	.986
TG (mmol/L)	1.69 ± 0.82	1.85 ± 1.68	0.72	.472
HDL (mm/L)	1.11 ± 0.28	1.11 ± 0.30	−0.18	.858
LDL (mmol/L)	2.96 ± 0.78	2.70 ± 0.81	−1.78	.078
FPG (mmol/L)	6.23 ± 1.21	5.73 ± 0.74	−2.72	.008
FT3 (pmol/L)	5.04 ± 0.57	4.82 ± 0.82	−1.78	.078
FT4 (pmol/L)	15.92 ± 1.78	16.11 ± 2.36	0.5	.618
TSH (mIU/L)	2.85 ± 2.24	1.99 ± 1.11	−2.61	.01
PLT (10^^9^/L)	232.88(182.5275.5)	218(188,270)	−0.46	.646
NC (10^^9^/L)	3.73 ± 1.44	3.23 ± 1.03	−2.17	.032
LC (10^^9^/L)	1.87 ± 0.74	1.90 ± 0.53	0.32	.753
NLR	2.42 ± 1.59	1.78 ± 0.62	−2.81	.006

ALT = alanine aminotransferase, AST = aspartate aminotransferase, FPG = fasting plasma glucose, FT3 = free triiodothyronine, FT4 = free thyroxine, HDL = high-density lipoprotein, LC = lymphocyte count, LDL = low-density lipoprotein, NC = neutrophil count, NLR = neutrophil to lymphocyte ratio, TC = total cholesterol, TG = triglyceride, TSH = PLTplatelet, γ-GT = γ-glutamyltranspeptidase.

Table 4 describes the comparison between the histopathological characteristics of the 2 groups of patients. We found that compared with the non-NASH group, the NASH group had relatively more severe steatosis, lobular inflammation, ballooning degeneration, and higher liver fiber staging (all *P* < .05).

**Table 4:** Histopathological characteristics between NASH and non-NASH groups.

Variables	NASH (N = 72)	Non-NASH (N = 55)	Statistics	*P* value
Steatosis			41.83	<.001
1	17 (23.6%)	43 (78.2%)		
2	32 (44.4%)	12 (21.8%)		
3	23 (31.9%)	0 (0%)		
Inflammation			53.4	<.001
0	0 (0%)	13 (23.6%)		
1	14 (19.4%)	31 (56.4%)		
2	36 (50%)	11 (20.0%)		
3	22 (30.6%)	0 (0%)		
Ballooning			27.01	<.001
0	0 (0%)	13 (23.6%)		
1	50 (69.4%)	39 (70.9%)		
2	22 (30.6%)	3 (5.5%)		
Fibrosis			41.16	<.001
0	0 (0%)	11 (20.0%)		
1	17 (23.6%)	23 (41.8%)		
2	34 (47.2%)	20 (36.4%)		
3	11 (15.3%)	1 (1.8%)		
4	10 (13.9%)	0 (0%)		

NASH = nonalcoholic steatohepatitis.

Among the study population included in this study, 51 cases (40.16%) were in the no/mild liver fibrosis group (F0-1) and 76 cases (59.84%) were in the significant liver fibrosis group (F2-4). The general clinical data characteristics of the 2 groups were described in Table 5. The age, BMI, AST, FPG, NLR, and TSH levels of patients with significant fibrosis were significantly higher than those of the control group. At the same time, there were 23 patients with diabetes in the significant liver fibrosis group. Cases (18.11%) were significantly higher than the mild liver fibrosis group, with statistical differences (all *P* < .05); while the 2 groups were in ALT, γ-GT, TC, TG, HDL, LDL, PLT, NC There was no significant difference in the levels of FT3, FT4 (all *P* > .05).

**Table 5:** Comparison between no/mild fibrosis group and significant fibrosis group.

Variables	No/Mild Fibrosis (N = 51)	Significant fibrosis (N = 76)	Statistics	*P* value
Age (yr)	40.25 ± 11.27	45.46 ± 15.39	−2.071	.04
Gender (M/ F)	23/28	36/40	0.063	.801
Hypertension (No/Yes)	38/13	49/27	1.733	.42
Diabetes (No/Yes)	49/2	53/23	16.487	<.001
BMI (kg/m^2^)	26.14 ± 3.19	27.43 ± 3.75	−2.015	.046
ALT (U/L)	72(23,115)	79(38.25,145)	−1.439	.15
AST (U/L)	38(21,60)	48.5(27,80)	−2.172	.03
γ-GT (U/L)	71.9(41,145)	72.5(45.25,126.25)	−0.098	.922
TC (mmol/L)	5.06 ± 1.01	4.90 ± 1.06	0.856	.393
TG (mmol/L)	1.68 ± 0.72	1.82 ± 1.53	−0.599	.55
HDL (mm/L)	1.08 ± 0.29	1.13 ± 0.29	−0.971	.334
LDL (mmol/L)	2.89 ± 0.78	2.82 ± 0.82	0.472	.638
FPG (mmol/L)	5.70 ± 0.57	6.22 ± 1.25	−2.797	.006
FT3 (pmol/L)	4.90 ± 0.71	4.98 ± 0.69	−0.622	.535
FT4 (pmol/L)	16.11 ± 1.93	15.93 ± 2.13	0.483	.63
TSH (mIU/L)	1.90 ± 0.79	2.87 ± 2.27	−2.936	.004
PLT (10^^9^/L)	232(204,276)	228.5(173.25,268.5)	−1.321	.187
NC (10^^9^/L)	3.34 ± 0.93	3.63 ± 1.49	−1.26	.21
LC (10^^9^/L)	2.03 ± 0.53	1.78 ± 0.71	2.106	.037
NLR	1.76 ± 0.81	2.40 ± 1.50	−2.763	.007

ALT = alanine aminotransferase, AST = aspartate aminotransferase, FPG = fasting plasma glucose, FT3 = free triiodothyronine, FT4 = free thyroxine, HDL = high-density lipoprotein, LC = lymphocyte count, LDL = low-density lipoprotein, NC = neutrophil count, NLR = neutrophil to lymphocyte ratio, TC = total cholesterol, TG = triglyceride, TSH = PLTplatelet, γ-GT = γ-glutamyltranspeptidase.

### 3.3. Difference of serum TSH and NLR level in NASH and fibrosis groups

**Figure 1.**
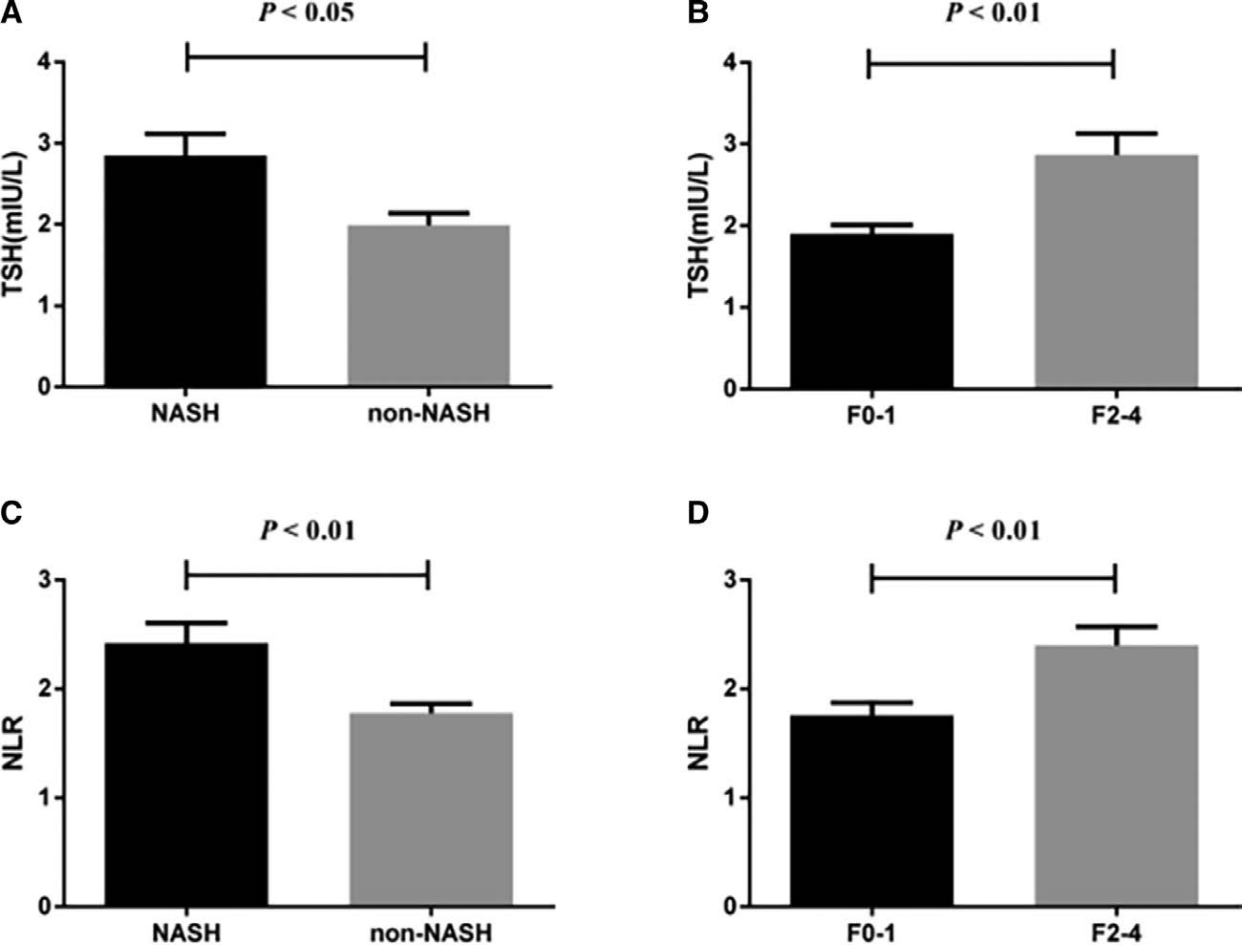
The level difference of TSH and NLR in NASH and fibrosis group. NASH = nonalcoholic steatohepatitis, NLR = neutrophil to lymphocyte ratio, TSH = thyroid stimulating hormone.

From the figure below, we can find that the TSH level of the NASH group was significantly higher than that of the non-NASH group; similarly, the TSH level of patients with significant liver fibrosis (F2-4) was significantly higher than that of the no/mild liver fibrosis group (F0- 1) (all *P* < .05), see Figure 1A and B TSH content in serum in NASH and fibrosis groups. In addition, the NLR value of the NASH group was significantly higher than that of the non-NASH group; similarly, the NLR value of patients with significant liver fibrosis (F2-4) was significantly higher than that of the no/mild liver fibrosis group (F0-1) (both *P* < .05), see Figure 1C and D, NLR content in serum in NASH and fibrosis groups.

**Figure 2.**
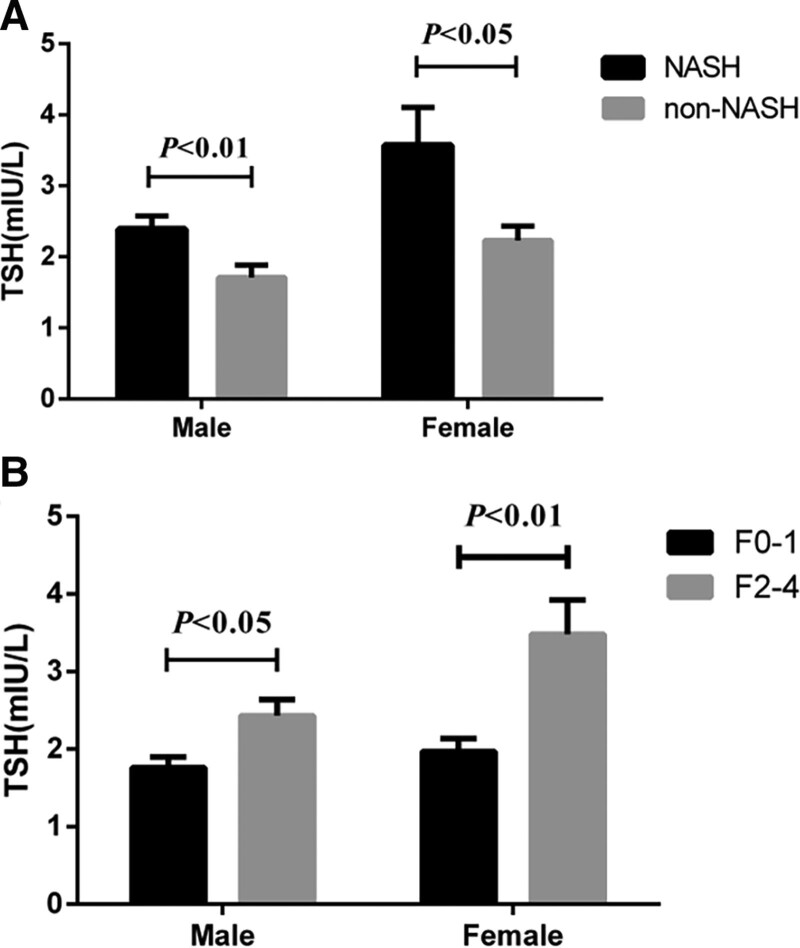
Subgroup comparison of TSH levels based on gender. TSH = thyroid stimulating hormone.

Because thyroid-related hormones are closely related to gender, we conducted a further gender-based subgroup analysis. The results showed that the TSH level of the NASH group was still higher than that of the non-NASH group, whether in the male subgroup or the female subgroup. There are statistical differences (all *P* < .05); similarly, the results of subgroup analysis show that the TSH level of the significant fibrosis group (F2-4) is still significantly higher than the no/mild fibrosis group (F0-1) (both *P* < .05), see Figure 2A and B serum TSH content of different genders.

Tables 3 and 5 reported that TSH and NLR were significantly associated with NASH and liver fibrosis, respectively. In order to exclude the influence of confounders, we further conducted the logistic regression. Given the gender-based grouping analysis has been conducted, gender didn’t enter into further model. Finally, we chose diabetes, DMI, ALT, AST, FPG, FSH and NLR for multivariate binary logistic regression analysis. As shown in Tables 6 and 7, a higher level of TSH and NLR was still independently associated with NASH and liver fibrosis significantly after controlling for other variates, respectively. In addition, we also found that diabetes was associated with NASH and liver fibrosis with statistical significance.

**Table 6:** Factors associated with multivariate regression of NASH.

Variable	Multivariate analysis	
	OR (95% CI)	*P* value
Diabetes	3.983 (1.004–15.806)	.049
BMI (kg/m^2^)	1.126 (0.972–1.305)	.114
ALT (U/L)	1.004 (0.998–1.011)	.191
AST (U/L)	1.005 (0.995–1.015)	.357
FPG (mmol/L)	1.124 (0.747–1.690)	.576
TSH (mIU/L)	1.402 (1.034–1.900)	.029
NLR	2.029 (1.251–3.289)	.004

ALT = alanine aminotransferase, AST = aspartate aminotransferase, FPG = fasting plasma glucose, NLR = neutrophil to lymphocyte ratio, TSH = thyroid stimulating hormone.

**Table 7:** Factors associated with multivariate regression of significant fibrosis.

Variable	Multivariate analysis	
	OR (95% CI)	*P* value
Diabetes	7.751 (1.330–45.181)	.023
BMI (kg/m^2^)	1.176 (0.999–1.384)	.051
ALT (U/L)	1.001 (0.995–1.007)	.742
AST (U/L)	1.006 (0.996–1.016)	.251
FPG (mmol/L)	1.667 (0.912–3.045)	.097
TSH (mIU/L)	2.775 (1.378–5.588)	.004
NLR	2.604 (1.265–5.359)	.009

ALT = alanine aminotransferase, AST = aspartate aminotransferase, FPG = fasting plasma glucose, NLR = neutrophil to lymphocyte ratio, TSH = thyroid stimulating hormone.

### 3.4. The diagnostic accuracy of TSH and NLR level in NASH and fibrosis

**Figure 3.**
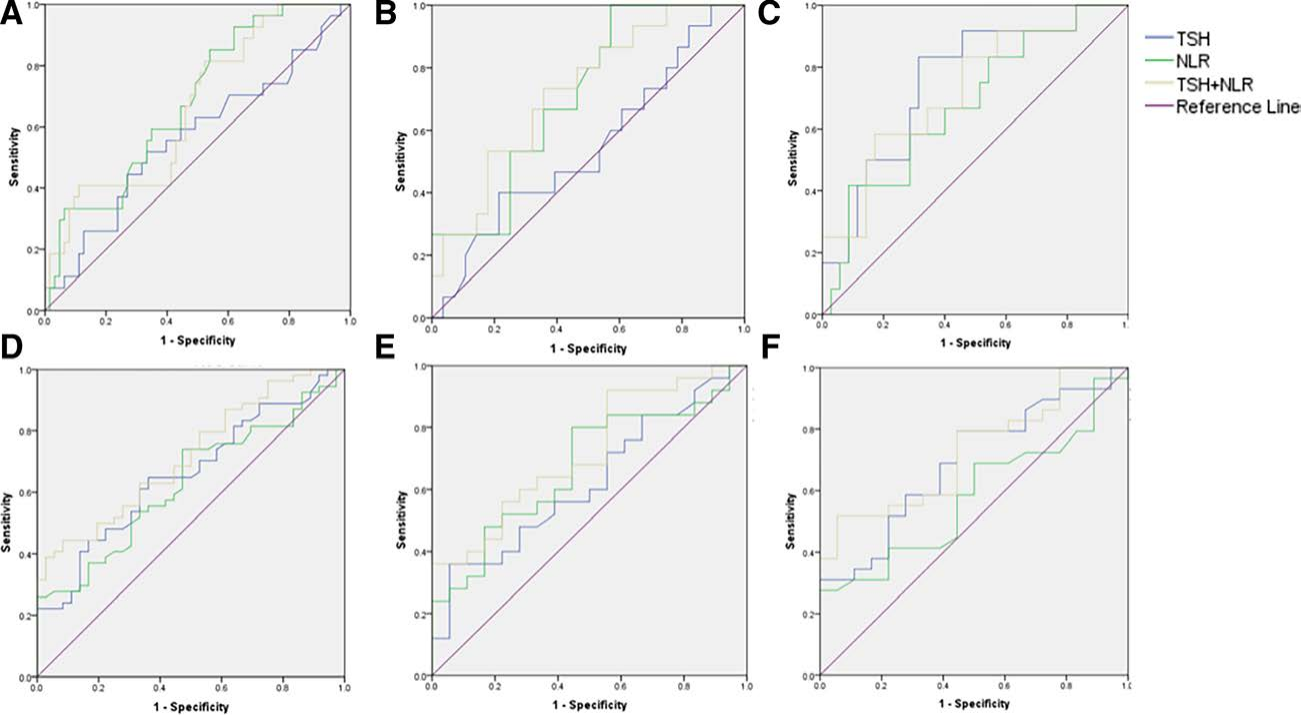
ROC curve of TSH, NLR and their combined indexes in diagnosis of NASH and liver fibrosis. NASH = nonalcoholic steatohepatitis, NLR = neutrophil to lymphocyte ratio, ROC = receiver operating characteristic, TSH = thyroid stimulating hormone.

The ROC curves of TSH, NLR and combination for predicting NASH and significant liver fibrosis are drawn, see Figure 3. The AUROC, Youden index, cutoff values, sensitivity, specificity are shown in Tables 8 and 9.

**Table 8:** ROC curve of TSH, NLR and their combined indexes in diagnosis of NASH.

	AUROC	95%CI	Youden index	Cutoff	Sensitivity (%)	Specificity (%)
Total						
TSH	0.564	0.455–0.668	0.1852	2.24	51.85	66.67
NLR	0.682	0.575–0.776	0.3122	1.65	85.19	46.03
TSH + NLR	0.664	0.544–0.783	0.2960	-	40.70	88.90
Male						
TSH	0.546	0.388–0.699	0.1857	1.26	40.00	78.57
NLR	0.711	0.544–0.783	0.4286	1.39	100.00	42.86
TSH + NLR	0.724	0.569–0.879	0.3760	-	73.30	64.30
Female						
TSH	0.755	0.607–0.868	0.5190	2.37	83.33	68.57
NLR	0.679	0.526–0.807	0.3310	2.63	41.67	91.43
TSH + NLR	0.729	0.563–0.895	0.4120	-	58.30	82.90

AUROC = the area under the receiver operating characteristic, CI = confidence interval, NLR = neutrophil to lymphocyte ratio, TSH = thyroid stimulating hormone.

**Table 9:** ROC curve of TSH, NLR and their combined indexes in diagnosis of liver fibrosis.

	AUROC	95%CI	Youden index	Cutoff	Sensitivity (%)	Specificity (%)
Total						
TSH	0.657	0.549–0.754	0.2870	1.80	64.81	63.89
NLR	0.630	0.521–0.729	0.2685	1.65	74.07	52.78
TSH + NLR	0.717	0.613–0.821	0.3610	-	44.40	91.70
Male						
TSH	0.623	0.463–0.766	0.3044	2.30	36.00	94.44
NLR	0.673	0.513–0.808	0.3556	1.65	80.00	55.56
TSH + NLR	0.720	0.568–0.872	0.3640	-	92.00	44.40
Female						
TSH	0.693	0.541–0.819	0.3487	1.8	79.31	55.56
NLR	0.581	0.429–0.724	0.2759	2.63	27.59	100.00
TSH + NLR	0.728	0.585–0.871	0.4610	-	51.70	94.40

AUROC = the area under the receiver operating characteristic, CI = confidence interval, NLR = neutrophil to lymphocyte ratio, TSH = thyroid stimulating hormone.

The AUROC of TSH, NLR and combination in diagnosis of NASH are 0.564 (0.455–0.668), 0.682 (0.575–0.776) and 0.664 (0.544–0.783). Considering the correlation between TSH and gender, we performed subgroup ROC analysis based on gender. In male patients, the AUROC were 0.546 (0.388–0.699), 0.711 (0.544–0.783) and 0.724 (0.569–0.879), while the values were 0.755 (0.607–0.868), 0.679 (0.526–0.807), and 0.729 (0.563–0.895) in female groups. It demonstrated that the combined application of TSH and NLR may be more indicative of NASH in male patients, while in female patients, TSH alone is more relevant to NASH. Although no significant difference was observed in those indices.

When TSH and NLR were used to predict liver fibrosis, the application of combination shows higher test accuracy in both male and female patients. The AUROC results of total patients, male patients and female patients were 0.657 (0.549–0.754), 0.623 (0.463–0.766) and 0.693 (0.541–0.819) in TSH; 0.630 (0.521–0.729), 0.673 (0.513–0.808) and 0.581 (0.429–0.724) in NLR; 0.717 (0.613–0.821), 0.720 (0.568–0.872) and 0.728 (0.585–0.871) in combination.

## 4. Discussion

Although several studies have shown that the probability of NAFLD in patients with hypothyroidism is 15.2% to 36.3%, the conclusions of these studies have not reached a consensus. In a meta-analysis of 14 observational studies, the researchers found no association among patients with hypothyroidism^[16]^; however, in another meta-analysis of 13 observational studies, the researchers found that the risk of NAFLD in patients with a 50% increase.^[17]^ We speculate that the different conclusions of the above studies may be due to the large heterogeneity of the included studies, such as differences in race, disease diagnosis methods, and disease severity of the research subjects. However, some recent studies have begun to focus on patients with NASH, but there is still controversy about the relationship between hypothyroidism and NASH. A study conducted by Liangpunsakul et al showed that the prevalence of hypothyroidism in NASH patients was significantly higher than that in the control group. Hypothyroidism and the occurrence of NASH are closely related, but this study is limited to not all patients with NASH have undergone liver biopsy. Some patients with NASH are diagnosed based on clinical and imaging; in addition, hypothyroidism in this study is diagnosed based on the history of TH replacement therapy, so there is a lack of relevant laboratory data for thyroid function testing^[15]^; similarly, another one A study found that under the background of the underlying disease of NASH, the risk of hypothyroidism is higher, but they also diagnose hypothyroidism based on the history of TH replacement therapy, and lack data related to thyroid parameters.^[9]^ On the contrary, in a retrospective clinical study from Brazil, which included 103 NAFLD patients diagnosed by liver biopsy, the researchers found that there was no connection between hypothyroidism and the progression of NAFLD. However, the diagnosis of hypothyroidism is based on the current history of thyroid drug replacement therapy, and there is also no detection of thyroid-related parameters such as FT3, FT4, and TSH.^[14]^ Researchers have found in rabbit animal models that hypothyroidism can cause moderately ill NASH.^[29]^ In terms of mechanism, patients with hypothyroidism have oxidative stress and lipid peroxidation.^[30]^ At the same time, studies have shown that hypothyroidism has a certain effect on mitochondrial function.^[31]^ More importantly, a recent study showed that oxidative stress and mitochondrial dysfunction are involved in the pathogenesis of NASH.^[1]^ Therefore, we speculate that thyroid-related hormones may participate in the development of NASH through oxidative stress and mitochondrial dysfunction.

Yan et al reported that the increase in serum TSH levels can cause the occurrence and development of NASH.^[32]^ Some studies have also shown that increased serum TSH levels are closely related to the occurrence of NASH.^[33,34]^ One possible explanation is that increased serum TSH levels are closely related to metabolic syndrome.^[35]^ A cross-sectional study showed that serum TSH levels can directly affect the lipid composition of liver cell membranes^[36]^ In terms of mechanism, in addition to indirectly exerting biological effects by influencing TH, elevated serum TSH can also directly act on TSH receptors on the surface of liver cell membranes, leading to up-regulation of the activity of sterol regulatory element binding protein 1c, which in turn causes NASH Occurrence and disease progression.^[32]^ The study is similar to our results. The results of this study show that there is a strong correlation between serum TSH levels and NASH. The average TSH level of the NASH group is significantly higher than that of the non-NASH group. More importantly, we found that in the logistic regression analysis, the correlation between serum TSH levels and NASH still exists and is not affected by other confounding factors. This means that for NAFLD patients, serum TSH levels can be used as an indirect marker to predict the occurrence of NASH.

The presence of significant liver fibrosis can predict the outcome of liver disease and assess the overall mortality of patients.^[37]^ A population-based prospective study from the Netherlands explored and confirmed the association between the severity of NAFLD and hypothyroidism. Researchers found that high levels of TSH and/or low levels of FT4 are closely related to increased risk of liver fibrosis.^[11]^ A cross-sectional study from South Korea found that hypothyroidism and liver fibrosis are related to a certain degree. The study included 425 patients with NAFLD diagnosed by liver biopsy. The researchers found that the severity of fibrosis and serum levels confirmed by liver biopsy The increase in TSH levels is closely related, and the correlation is not affected by other confounding factors.^[12]^ However, none of the above studies in the Netherlands and South Korea detected the FT3 level of the subjects studied. The above findings are similar to our results. The results of this study found that the TSH level of the significant fibrosis group was significantly higher than that of the no/mild fibrosis group, indicating that there is a certain correlation between the increase in TSH and moderate to severe fibrosis. It may be used as an independent factor of fibrosis aggravation. A recent study by Alonso-Merino et al pointed out that mice lacking TRs spontaneously accumulate collagen in the liver, and when T3 is administered, it inhibits carbon tetrachloride-induced liver fibrosis. At the same time, they proposed that TRs bind to T3. It can directly antagonize the TGF-β signal by reducing Smad phosphorylation and preventing Smads from recruiting to the TGF-β target gene promoter.^[38]^

To date, only a few studies have explored the role of NLR in assessing the histological severity of fatty liver disease. However, there are still controversies between the studies, and the role of NLR in predicting NASH and liver fibrosis is still unclear. Alkhouri et al have reported that compared with non-NASH patients, NLR is positively correlated with the occurrence of NASH. In addition, NLR is closely related to each component of the NAFLD activity score (steatosis, lobular inflammation, and ballooning) and the stage of liver fibrosis.^[39]^ Two other recent studies have reported similar results, and studies have shown that NLR is significantly associated with NASH and the degree of liver fibrosis.^[40]^ In contrast, a recent study by Kara et al explored this correlation and reported that NLR was not associated with the degree of histological inflammation and fibrosis in NAFLD patients. Therefore, NLR cannot be used as a noninvasive surrogate for the degree of NASH and liver fibrosis.^[41]^ However, one of the main findings of our study is that the NLR level of the NASH group was significantly higher than that of non-NASH patients, and the NLR level of the moderate to severe fibrosis group was significantly higher than that of the mild fibrosis group, which is consistent with our initial hypothesis. In addition, in multiple regression analysis, this correlation still exists and is independent of other confounding factors. This indicates that NLR has a significant correlation with NASH and significant liver fibrosis. NLR can clarify the persistent systemic inflammatory response in NAFLD, which can be used as a noninvasive index to predict the severity of NAFLD disease.

As for the relationship between TSH and NLR, few studies have explored the correlation between them in patients with fatty liver. A study conducted in obese children pointed out that TSH and NLR were both related to BMI, suggesting that they could reflect the inflammation degree of the body, but spearman analysis showed no statistical significance between them.^[42]^ In our study, there was no direct confirmation of the correlation between them, but TSH and NLR were significantly increased in both NASH and moderate to severe liver fibrosis groups. Based on this point, we further performed the ROC curve. When judging moderate and severe liver fibrosis, the combination of the 2 indices can achieve higher accuracy. However, when judging NASH, the relationship between TSH and NASH in female patients is loser than NLR or the combination of the two. Considering the relationship between thyroid function and gender, most of the women included in this article are premenopausal. Thus, the estrogen level may have some impact on the results, but the specific mechanism still needs further study.

Of course, our article has several limitations. Firstly, in a retrospective study, we inevitably have bias and partial data loss. Secondly, the sample size is too small. The inclusion criteria of this article were patients with NAFLD confirmed by pathology. Patients are always unwilling to accept a traumatic examination, especially those with simple NAFLD. Finally, we only selected common indicators such as TSH and NLR. It’s necessary to carry out more research on thyroid function indicators such as T3 and T4. In conclusion, we still need large samples and multicenter prospective studies to confirm the application of TSH and NLR in liver diseases.

## 5. Conclusions

In this study, serum TSH and NLR levels of NASH and moderate to severe fibrosis patients were higher than those of the corresponding control group, and in the analysis of subgroups of different genders, differences in serum TSH levels between the 2 groups still exist. Our research shows that serum TSH levels and NLR are closely related to the severity of NAFLD disease, suggesting that this simple and easily accessible laboratory index may be included in future non-invasive diagnostic scoring models to determine the occurrence of NASH and the degree of fibrosis make predictions.

## Author contribution

L. Liu drafted the manuscript and statistical analysis; P. Li the concept, design of the study; B. Niu, J. Feng, P. Zhang, X. Wu, WK. Chu the data acquisition; YQ. Mi, YG. Liu the critical revision for intellectual content. All authors critically revised the manuscript, approved the final version to be published, and agree to be accountable for all aspects of the work.

**Data curation:** YongGang Liu, Jing Feng, Peng Zhang, Xue Wu, WeiKe Chu.

**Investigation:** Bin Niu.

**Methodology:** Ping Li.

**Writing – original draft:** Liang Liu.

**Writing – review & editing:** Ping Li, YuQiang Mi.
